# Economic evaluation of expanding inguinal hernia repair among adult males in Ghana

**DOI:** 10.1371/journal.pgph.0000270

**Published:** 2022-04-04

**Authors:** Zin Min Thet Lwin, Birger Forsberg, George Keel, Jessica H. Beard, Joachim Amoako, Michael Ohene-Yeboah, Stephen Tabiri, Jenny Löfgren

**Affiliations:** 1 The Swedish Institute for Health Economics, Lund, Sweden; 2 Department of Global Public Health, Karolinska Institutet, Stockholm, Sweden; 3 Department of Learning, Informatics, Management and Ethics, Karolinska Institutet, Stockholm, Sweden; 4 Department of Surgery, Temple University Lewis Katz School of Medicine, Philadelphia, PA, United States of America; 5 Department of Surgery, School of Medicine and Dentistry, University of Ghana, Accra, Ghana; 6 Department of Surgery, School of Medicine, University for Development Studies and Tamale Teaching Hospital, Tamale, Ghana; 7 Department of Molecular Medicine and Surgery, Karolinska Institutet, Stockholm, Sweden; Children’s Hospital of Eastern Ontario, University of Ottawa, CANADA

## Abstract

An unmet need for inguinal hernia repair is significant in Ghana where the number of specialist general surgeons is extremely limited. While surgical task sharing with medical doctors without formal specialist training in surgery has been adopted for inguinal hernia repair in Ghana, no prior research has been conducted on the long-term costs and health outcomes associated with expanding operations to repair all inguinal hernias among adult males in Ghana. The study aimed to estimate cost-effectiveness of elective open mesh repair performed by medical doctors and surgeons for adult males with primary inguinal hernia compared to no treatment in Ghana and to project costs and health gains associated with expanding operation services through task sharing between medical doctors and surgeons. The study analysis adopted a healthcare system perspective. A Markov model was constructed to assess 10-year differences in costs and outcomes between operations conducted by medical doctors or surgeons and no treatment. A 10-year budget impact analysis on service expansion for groin hernia repair through increasing task sharing between the providers was conducted. Incremental cost-effectiveness ratios for medical doctors and surgeons were USD 120 and USD 129 respectively per disability-adjusted life year (DALY) averted compared to no treatment, which are below the estimated threshold value for cost-effectiveness in Ghana of USD 371–491. Repairing all inguinal hernias (1.4 million) through task sharing between the providers in the same timeframe is estimated to cost USD 194 million. Total health gains of 1.5 million DALYs averted are expected. Inguinal hernia repair is cost-effective regardless of the type of surgical provider. Scaling up of inguinal hernia repair is worthwhile, with the potential to substantially reduce the disease burden in the country.

## Introduction

Inguinal hernia is one of the most common surgical conditions worldwide, affecting 220 million people [1,2]. Over 40,000 people die from complications from inguinal hernias and about 3,500,000 disability-adjusted life years (DALYs) are lost annually [2]. Inguinal hernia repair is one of the essential surgical procedures prioritized by the World Health Organization (WHO) [3]. Each year, approximately 20 million hernia repairs are performed [2,4], but this accounts for less than one-tenth of global hernia prevalence. In sub-Saharan Africa (SSA) where hernia prevalence is estimated to be the highest [2], one of the main causes of this unmet surgical need is a significant shortage of surgeons in the region [5].

In Ghana, the prevalence of inguinal hernia is 13% and untreated inguinal hernia is 10.8% among the adult male population [6]. This high prevalence along with a very low hernia repair rate of approximately 30–65 per 100,000 population results in an estimated backlog of about one million inguinal hernias over 10-years [7–10]. Currently, the majority (about 74%) of hernia patients undergo operations at district hospitals of which 38% do not have surgeons [9] and where priority is given to emergency cases. Hence, there is a need to increase elective hernia repair, particularly among adult males, to reduce the disease burden in the country.

To bolster elective hernia repair rates, the WHO promotes task sharing of human resources in low-resource settings [11]. Task-sharing is defined as the pragmatic transfer of tasks from highly qualified professionals, in this case qualified surgeons, to those with shorter training periods or fewer qualifications, with sharing responsibility for high-quality outcomes [12]. Surgical task sharing between specialist surgeons, non-specialist medical doctors (MDs) as well as associate clinicians has shown promising results in sub-Saharan Africa [13–15].

The long-term costs and health outcomes of the potential scale-up of surgical care for inguinal hernia in Ghana has not been investigated previously. This study aimed to estimate cost-effectiveness from a healthcare system perspective of elective open mesh repair for adult men with symptomatic primary inguinal hernia compared to no treatment in Ghana, and project costs and health gains associated with expanding the number of elective operations provided. The health system challenges leading to the shortage in hernia repairs are complex in Ghana [7]. Task sharing can be one part of the solution. However, given the WHO’s recommendations [3], the potential impact of task sharing between MDs and surgeons on cost-effectiveness is explored. Filling this knowledge gap will contribute to building evidence that will inform decisions on addressing the unmet repair needs of inguinal hernia patients, particularly adults.

## Materials and methods

### Definitions

**Surgeon**—specialist general surgeon

**Medical Doctor (MD)**—medical doctor without formal specialist training in surgery. For these surgical providers, training to provide surgical services is done on the job during the two years following their medical education.

**Associate Clinician (AC)**—mid-level healthcare provider (non-doctors) with or without formal surgical training.

### Data sources

The study combined data from previous studies in Ghana, including a prospective cohort study on outcomes of 242 patients after inguinal hernia repair with mesh conducted by MDs and surgeons from February 2017 to September 2018 [14] and a prevalence study of inguinal hernia among adult male populations in the Ashanti Region in 2015 [6]. The Temple University Institutional Review Board (Temple IRB number: 25359) and the Ghana Health Service Ethical Review Committee (GHS-ERC:01/10/16) approved this study.

### Model structure

**Fig 1:**
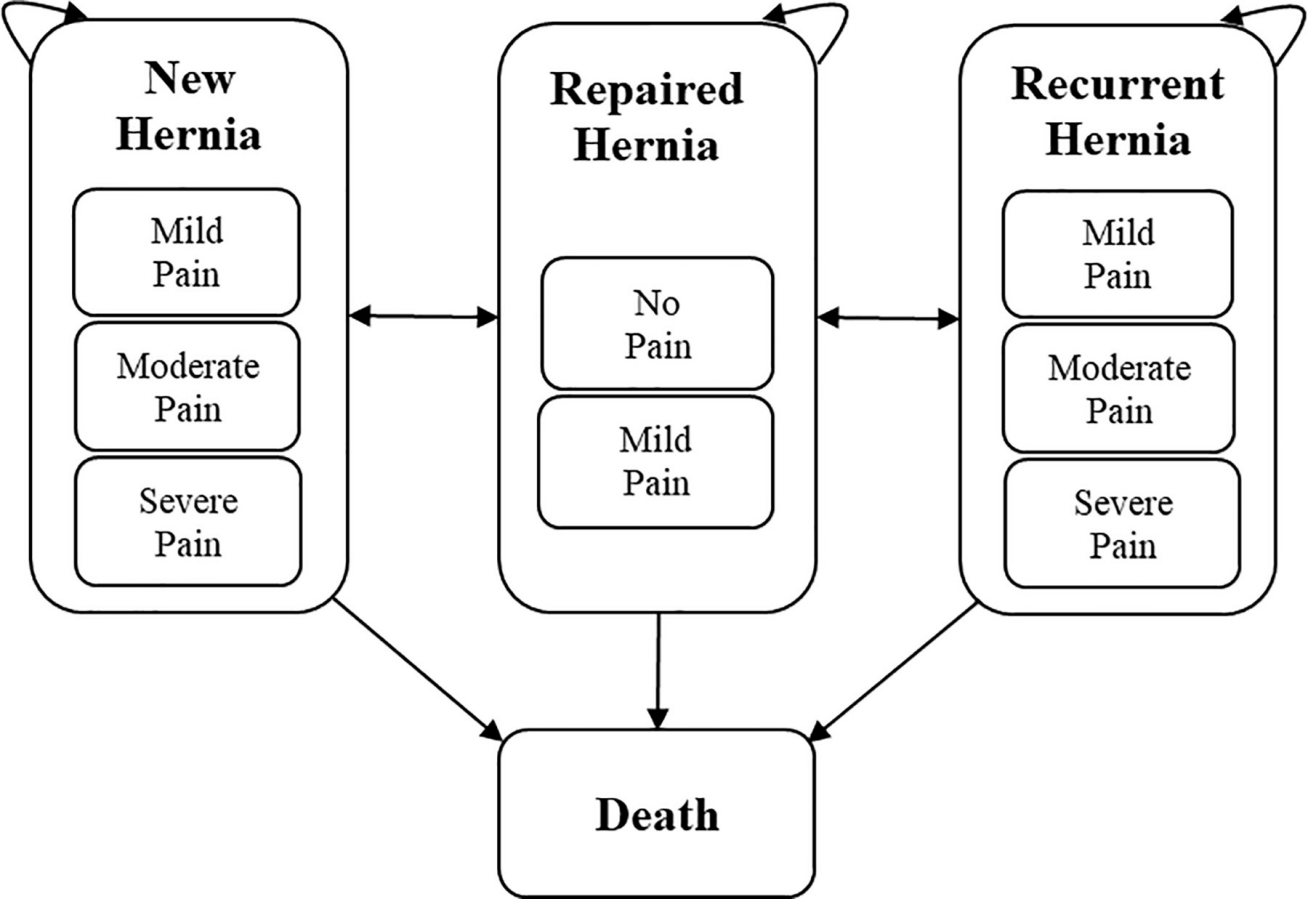
Markov model. All individuals simulated in the model start in the ‘New Hernia’ state in cycle zero. Depending on the user-defined rate of hernia repair (100% in the base-case analysis and starting from the current rate of 1.57% [9] and gradually expanded in the budget-impact analysis), patients can remain or move to the ‘Repaired Hernia’ state. Patients can then remain or move to the ‘Recurrent Hernia’ state based on the risk of recurrent hernia [26] or return to the ‘New Hernia’ state based on the risk of contralateral hernia [1]. Probabilities of having different pain levels in each health state were taken from the previous cohort study [14]. Transitions among the pain levels within each health state were not considered as the model only considers the presenting pain of the patients upon seeking care. Patients can move from any health state to the ‘Death’ state in each cycle. These transitions are based on age-dependent all-cause mortality risks according to the WHO’s life table for Ghana in 2016 and reported pre-mature mortality risks without surgery [19,20,23]. The ‘Death’ state is an absorptive state in the model where simulated patients accumulate.

A Markov model was built in Microsoft Excel to fit the decision problem. Such a model allows for the conceptualization of the clinical course of inguinal hernia in which individuals experience a set of health states without interactions between individuals. The model was structured to capture the care process of the condition within the time horizon and included six mutually exclusive health states and transitions among these states (Fig 1). The representation of clinical care processes was developed with the support of the clinical experts within the team. The health states involved cycles where individuals have new (including contralateral), repaired or recurrent inguinal hernia. All living health states were sub-categorised into different levels of pain. The final state represents death as an absorbing health state. The model’s cycle length is one year.

### Effectiveness

The primary health outcome measure of the study was disability-adjusted life years (DALYs) which is widely used to measure the burden of disease, particularly in LMICs [2]. In terms of DALY values, a value of 0 is assigned for no disability and 1 for the worst possible disability, which in turn is equivalent to the loss of one year of life with full health. No age and time weighting were applied in line with the recommendation from the Global Burden of Disease Study 2010 [16]. Formulas used for calculation of DALY are shown below:

DALY = years lived with disability (YLD) + years of life lost (YLL)

YLD = disability weight (DW) × remaining life expectancy at time of surgery

YLL = risk of early death without surgery × remaining life expectancy at time of surgery

In a previous prospective cohort study, outcomes on recurrence, patient satisfaction, pain and self-assessed health status at one year after open mesh repair were compared between MD and surgeon groups, and no significant difference was found [14]. Degrees of pain suffered by patients, immediately before and one year after surgery, were collected using the Inguinal Pain Questionnaire (IPQ) [14] which is a validated disease-specific instrument for measuring chronic pain after inguinal hernia repair [17]. For the current analysis, the IPQ scores were converted into respective levels of DW, according to the Global Burden of Disease 2017 Study [18] as shown in Table 1. Life expectancy was taken from the WHO’s life table for Ghana in 2016.

**Table 1:** Conversion of Inguinal Pain Questionnaire (IPQ) Score to Disability Weight (DW) according to the Global Burden of Disease 2017 study.

Description of Pain	IPQ Score	DW
No pain	1	0.0
Mild pain		
Pain present but could easily be ignored	2	0.011
Pain present could not be ignored, but did not interfere with everyday activities	3	0.011
Moderate pain		
Pain present could not be ignored, but interferes with concentration on chores and daily activities	4	0.114
Pain present, could not be ignored, interferes with most activities	5	0.114
Severe pain		
Pain present could not be ignored, necessitated bed rest	6	0.324
Pain present could not be ignored, prompt medical advice sought	7	0.324

Risk of premature death without surgery was estimated based on the risk of having complications, such as incarceration or strangulation, and the mortality risk from these complications. The risk of having complications was approximated to be between 0.0018 and 0.0056 per patient-year based on earlier studies [19,20]. The latter was used in this study because the mean age of people with inguinal hernia was under 65 years and a higher risk of complications was expected in the age group. The mortality risk among people with complicated hernias was conservatively assumed to be 20 per cent as previous studies estimated the mortality risk in emergency hernia repair in sub-Saharan Africa to be over six percent. It can reach up to 22 percent if access to care is delayed by more than 72 hours [21,22] and 100 percent if no treatment is received [23].

### Costing

Direct costs associated with standard resource consumption for mesh hernia repair performed by an MD and a surgeon in Ghana were taken from a previous study by one of the authors [24] and used in the Markov model. The estimate included costs of medicines and materials, staff time, overhead costs, and capital costs at the Volta Regional Hospital, which is a public hospital in Ghana (Table 2). These data were collected from 2017–2018. The exchange rate at the time of the operations in 2017 was 4.5 Ghanaian cedi to 1 US dollar, and year-on-year inflation rates on health in Ghana from the World Bank’s data source [25] were applied to convert figures to 2020 prices.

**Table 2:** Resource use and prices included in the study.

Resource	Price in 2020 value (USD)
Medicines and materials used per operation	
Medicines	17.06
Materials	9.18
Mesh	13.20
Staff costs based on salaries per minute	
Medical doctor	14.54
Surgeon	23.85
Nurse	6.26
Overhead cost per operation	48.60
Capital costs per operation	
Operation room	2.23
Patient ward	6.67
Equipment	5.64

*Medicines* mainly include local anesthetics; *Materials* include personal protective equipment, blade, skin prep, gauze, syringes, hypodermic needles, suture, intravenous tubing and dressings.

In the previous study, cost items were broken down and calculated systematically. Medicines and materials were standardized for all patients. Synthetic polypropylene mesh was used in all operations. It was a locally available, commercial mesh, with the price of 11 USD per piece. The number of items required for a standard operation, together with market price of each item, were used in cost calculation. Staff costs were measured based on a standard surgical productivity rate of 5 hernia repairs per surgeon per day and average staff salaries: a surgeon or a medical doctor, an operating room nurse and other ancillary staff. The data on salaries were obtained from the hospital’s payroll for the 2017 year.

Overhead costs for operating rooms and patient wards were computed from room size as a proportion of hospital’s size, annual expenditures of the hospital and the average number of patients per day. These costs were obtained from the Volta Regional Hospital in Ho, Ghana which was the setting of the previous study. Annual expenditures were collected from the hospital’s annual budget report for the year 2017. Capital costs were calculated for operating rooms and patient wards by using square feet of the rooms and rental cost for similar office space in Ghana and for equipment by using market prices for buying new equipment. The capital costs were divided by the number of patients or operations per day. Depreciation was applied for equipment for the total duration of their use.

### Base-case analysis

The base-case analysis was conducted by using input values, listed in Table 3, to compare mesh repair performed by either MDs or surgeons with no treatment. Long-term costs and effects of treatment and no-treatment over a 10-year period were estimated by running a Markov model, and an incremental cost-effectiveness ratio (ICER) was calculated. The selected time horizon reflects the long-term costs and effects of the condition and operation. The time horizon was also in line with the duration of previous studies on risks of recurrent and contralateral hernias [1,26].

**Table 3:** Model inputs in the base-case setting.

Model inputs	Values	Sources
Starting age (years)	50	
Time horizon (years)	10	
Cost per procedure (2020 values in USD)		
Medical doctor group	123.40	
Surgeon group	132.71	
Risk of recurrent hernia	0.00376	[26]
Risk of contralateral hernia	0.00380	[1]
Risk of premature death without surgery	0.00112	[19,20,23]
Discount rate	3%	
Half-cycle correction	Yes	

### Sensitivity analysis

One-way deterministic sensitivity analyses (DSA) were performed by varying model inputs by 20% and imposing changes on starting age (from 50 to 60 years), discount rate (from 3% to 3.5%) and half cycle correction (from being applied to not being applied). A variation of ±20% within DSA is a common practice in modelling [27], as were the values used for discount rates. DSA input adjustments are presented in Table 4.

**Table 4:** Values used in one-way deterministic sensitivity analysis.

Parameters	Base-case value	Low Value	High value
Cost per procedure (±20%)			
Medical doctor group	123.40	98.72	148.07
Surgeon group	132.71	106.16	159.25
Disability weight (±20%)			
Mild pain; IPQ 2–3	0.01100	0.00880	0.01320
Moderate pain; IPQ 4–5	0.11400	0.09120	0.13680
Severe pain; IPQ 6–7	0.32400	0.25920	0.38880
Risk of recurrent hernia (±20%)	0.00376	0.00301	0.00451
Risk of contralateral hernia (±20%)	0.00380	0.00304	0.00456
Risk of premature death without surgery (±20%)	0.00112	0.00090	0.00134

Probabilistic sensitivity analysis (PSA) was also conducted using Monte Carlo simulation, where parameters were varied according to a probability distribution function (PDF) based on respective mean and standard deviation values obtained from previous literatures [1,18,26] as in Table 5. Variables with no available PDF were not included in the PSA. Simulations of 1,000 iterations were run for the analysis.

**Table 5:** Values used in probabilistic sensitivity analysis.

Parameters	Base-case value	PSA value*	Normal distribution function	
			Mean	Standard deviation
Disability weight				
Mild pain; IPQ 2–3	0.01100	0.01015	0.01140	0.00400
Moderate pain; IPQ 4–5	0.11400	0.09330	0.11400	0.02000
Severe pain; IPQ 6–7	0.32400	0.35332	0.32400	0.05500
Risk of recurrent hernia	0.00376	0.00389	0.00376	0.00013
Risk of contralateral hernia	0.00380	0.00202	0.00380	0.00073

*PSA* probabilistic sensitivity analysis.

*Values were varied in each simulation on account of the distribution function with respective mean and standard deviation.

### Budget impact analysis

The budget impact analysis was constructed to estimate the budget impact of reducing or eliminating the accumulated backlog of inguinal hernias among Ghanaian men [6,8], including additional ongoing incident cases by 2030. The analysis was implemented according to two scenarios where the current hernia repair rate in Ghana [9] was increased each year by increasing the number of MDs available to treat hernia. Each scenario took account of potential financial constraints in supporting the service expansion for inguinal hernia repair.

■ Scenario 1 involved a financially conservative approach with an annual budgetary cap of USD 10 million.
■ Scenario 2 involved a financially aggressive approach with a steady budget increase, reducing the number of untreated hernias by approximately 70% by 2025, and eliminating the backlog by 2030.

The direct costs associated with mesh hernia repair included the costs of medicines and materials, staff time, overhead and capital costs at the health facilities. Apart from direct costs, the cost of surgical training for MDs and facilities’ renovation was added to the analysis. Considering a 6-day hands-on training module at a hospital in Ghana incorporating the cost of travel and accommodation of trainers and trainees, training venue and equipment, the cost per trainee was estimated to be USD 2,000 by a clinical expert working on this project. Based on a previous study which reported the cost of building improvements, facility renovation and/or maintenance for performing hernia repairs in Ghana [28], a start-up cost of USD 18,000 was approximated after applying an inflation rate adjusting to a 2020 value.

Accounting for the overall workload of surgical providers, including MDs and surgeons, at district hospitals in Ghana, one provider was assumed to perform an average of 50 hernia repairs per year [9]. On average, 2 surgical providers who are skilled in providing the necessary surgical care are available per hospital [29]. These figures were included in the first scenario and the average productivity of each provider was increased to 200 hernia repairs per year in the second, where providers would likely spend a large proportion of their time performing hernia repair.

### Budget impact sensitivity analysis

One-way DSAs were conducted on the budget impact model by imposing ±20% variations on training and facility renovation costs and increasing the number of providers per facility and their average annual hernia repair. The impacts on the backlog of untreated hernias among Ghanian men by 2030 in scenario 1 and the cumulative 10-year budget in scenario 2 were assessed.

### Model validation

Fitness of the model was assessed by validating model parsimony and clinical relevance. Face validity and verification were conducted based on their feasibility and appropriateness within the study.

## Results

### Base-case analysis

The analysis shows that the incremental costs per DALY averted (ICER) from providing mesh repair by MDs and surgeons for adult men with symptomatic inguinal hernia compared to no treatment in Ghana are USD 120.09 and 129.15 respectively (Table 6). The ICER value associated with mesh repair by MDs when compared to surgeons shows a slightly less cost (average cost of USD 130.56 in the MD group vs. USD 140.41 in the surgeon group) for the same level of incremental health gain (average 1.08 DALYs averted) between the two groups.

**Table 6:** Cost-effectiveness analysis of mesh repair by different providers in Ghana (n = 707,121).

Interventions	Total cost in million (USD)	Total DALYs	Incremental cost in million (USD)	Incremental DALYs averted	ICER
No hernia repair		870,469.63			
Hernia repair by surgeons	99.29	101,718.64	99.29	768,750.98	129.15
Hernia repair by MDs (vs. no hernia repair)	92.32	101,718.64	92.32	768,750.98	120.09
Hernia repair by MDs (vs. surgeons	92.32	101,718.64	-6.97	0	Dominates*

*MDs* medical doctors; *DALY* disability-adjusted life year; *ICER* incremental cost-effectiveness ratio.

*The result needs to be taken with caution as the cost difference between the two groups is minor.

### Sensitivity analysis

**Fig 2:**
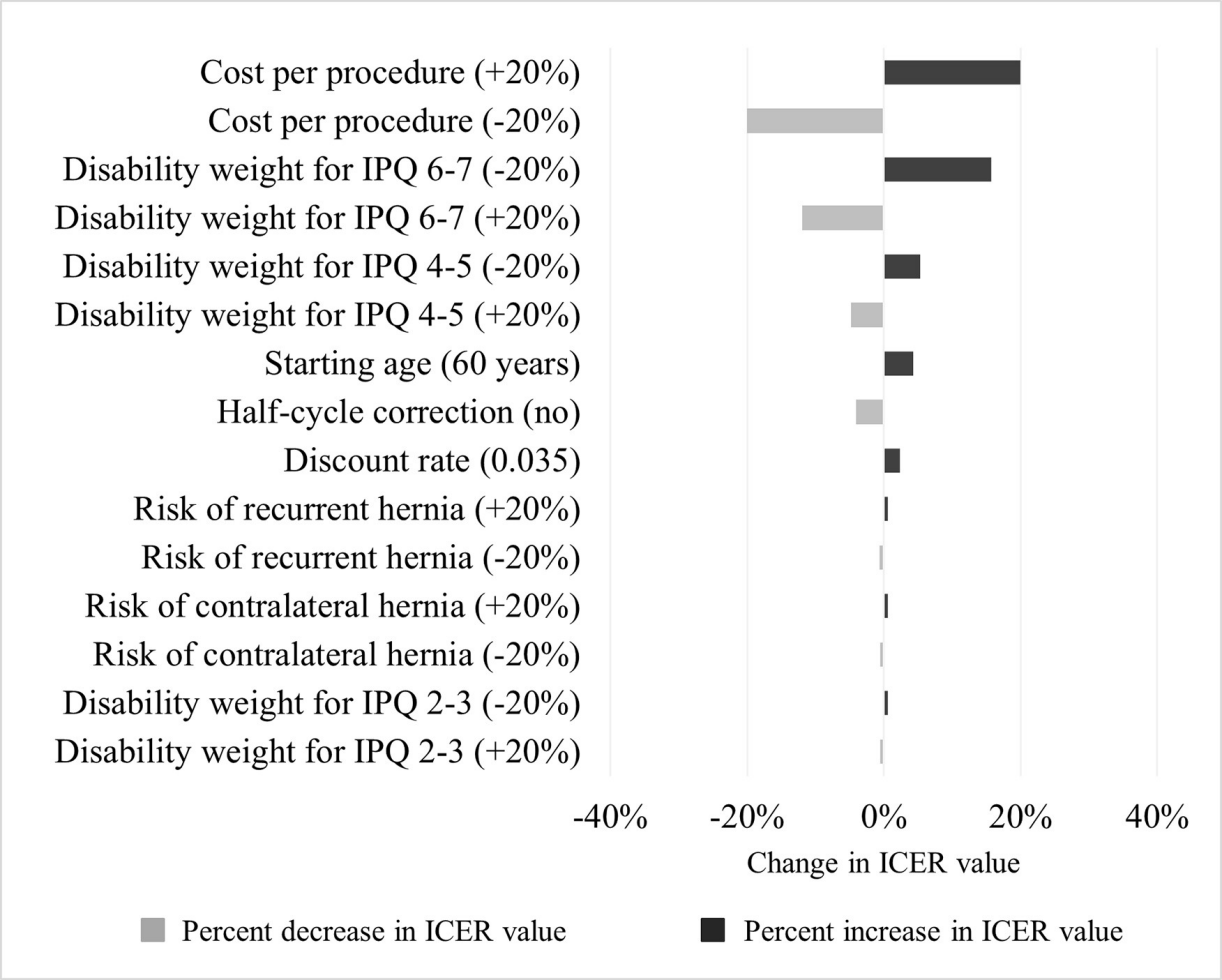
Deterministic sensitivity analysis of incremental cost-effectiveness ratios (ICERs). The tornado chart displays percent decrease or increase in ICER value from variations of inputs to the model.

One-way deterministic sensitivity analysis shows that the base-case value of the ICER for conducting mesh repair by either MDs or surgeons among symptomatic adult male patients in Ghana is robust to plausible variations of parameters and inputs as displayed in Fig 2. The most significant changes in the ICER are caused by the cost per procedure and the disability weight for severe pain level (IPQ 6–7). The risk of premature death without surgery, as it was minimal with inguinal hernia, made no change in the ICER value and therefore was excluded from the figure.

**Fig 3:**
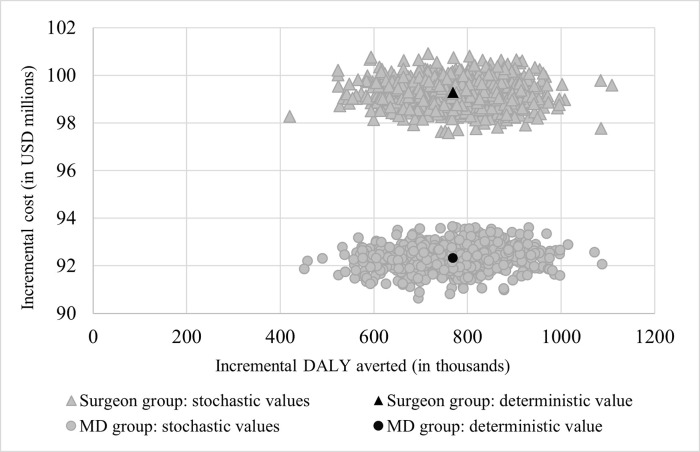
Stochastic sensitivity analysis of incremental cost-effectiveness ratios (ICERs). *MDs* medical doctors; *DALY* disability-adjusted life year. The scatter plot displays deterministic and stochastic values of incremental cost (in USD millions) and incremental DALYs averted (in thousands) in surgeon and MD groups from variations of inputs to the model.

**Fig 4:**
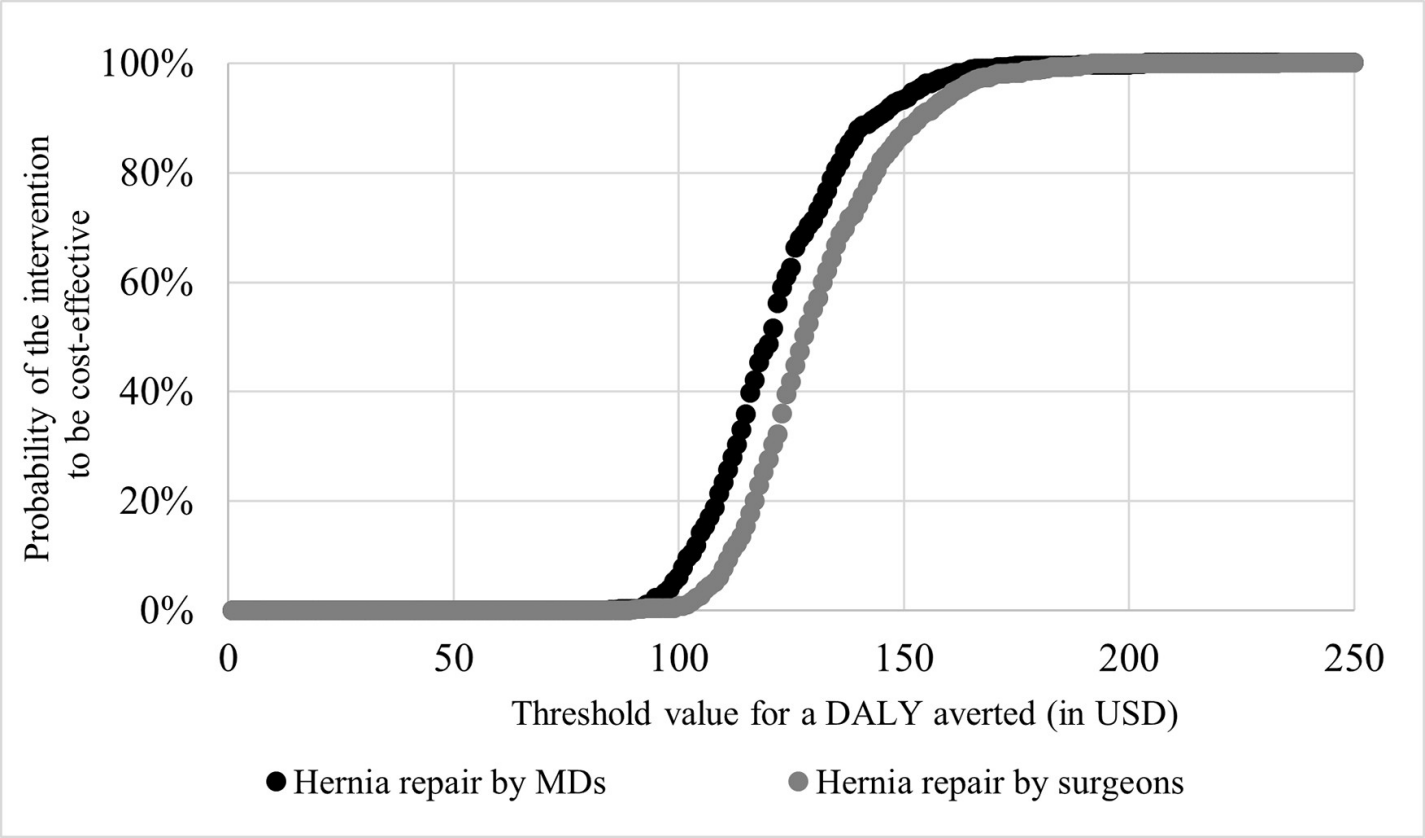
Cost-effectiveness acceptability curve of inguinal hernia repair by different providers in Ghana. *MDs* medical doctors; *DALY* disability-adjusted life year. The line graph displays probability of inguinal hernia repair, by MDs and surgeons, to be cost-effective by different threshold values for a DALY averted (in USD).

Likewise, probabilistic sensitivity analysis displays the resilient nature of the cost-effectiveness value to simultaneous variation of relevant parameters as can be seen in Fig 3. The figure also illustrates a similar but substantial reduction in DALYs in both groups while a lower incremental cost is associated with the operation performed by MDs. The cost-effectiveness acceptability curve in Fig 4 demonstrates that at WTP values of USD 125 and more per DALY averted, mesh repair by both providers is likely to be cost-effective compared to no treatment but with a slightly higher probability in the MD group than the surgeon group.

### Budget impact analysis

In the first scenario of service expansion with an annual budget ceiling of USD 10 million, a total of 752,000 inguinal hernias could be repaired, reducing the backlog among adult males in Ghana from about 1 million in 2020 to 733,000 in 2030 (S1 Fig). An additional 1,250 providers would need to be trained on the operation over the first five years. Total 795,000 DALYs can be expected to be averted, with an initial rapid increase in health gains followed by a steady amount over the remaining years (S2 Fig).

The aggressive budgetary approach with the providers’ increased productivity in the second scenario can be expected to treat 70% of untreated hernias by 2025 and eliminate the whole backlog of 1.4 million by 2030 (S3 Fig). An additional 1,200 providers would be required over the first three years. A total cost of USD 194 million is estimated for an expected health gain of 1.5 million DALYs averted. Annual cost and health gains are projected to escalate initially and then decline in the subsequent years (S4 Fig).

### Budget impact sensitivity analysis

Deterministic sensitivity analysis on the budget impact model illustrates that the remaining backlog of untreated hernias by 2030 in scenario 1 and the total estimated budget to address the entire backlog by 2030 in scenario 2 are significantly influenced by variations in annual hernia repair and number of providers in each facility, followed by facility renovation cost. The impact from training cost is less (S5 and S6 Figs).

## Discussion

This study aimed to estimate the cost and health effects from a national-level expansion of open mesh repair among adult males with symptomatic inguinal hernia in Ghana. There is a high probability that the operation is cost-effective by either MDs or surgeons as compared to no treatment in the study setting. The calculated ICERs are significantly lower than the country’s threshold values of USD 371–491 estimated by Ochalek et al. 2018 using different approaches to estimating health opportunity costs [30]. Comparison against ICERS identified within other prioritized healthcare activities, such as elimination of mother-to-child HIV transmission (USD 251–7,924) [31–34], vaccination against cholera and typhoid (USD 2,018) [35,36], and rural water supply or sanitation in low-income countries (2,200 USD) [37], also shows the operation is cost-effective. The results are robust to various sensitivity analyses with estimated willingness-to-pay values of USD 125 and more for the operation. Thus, the substantial reductions in burden of disease with sufficient investment of resources would be money well spent.

Between the two types of providers, expected levels of health gains are alike and there is no evidence to claim any significant difference in health outcomes, or lack thereof, after operations. However, for most elective hernia repairs, similar competency can be expected from both cadres after proper training. MDs performing operations is associated with slightly less cost when compared to surgeons, mainly because of salary differences, which results in the dominant value of ICER for MDs. However, the interpretation of this result needs to be taken with caution as the cost difference observed in the analysis is subtle and there is also a need of observing long-term health outcomes of the operations performed by these providers.

In Ghana, the human-resource gap is one of the long-standing barriers to surgical care [7]. Currently task sharing with medical doctors is practiced in order to expand the surgical workforce, and similar clinical outcomes after inguinal hernia repair performed by surgeons versus MDs was recently demonstrated in a prospective cohort study [14]. In order to meet the need for inguinal hernia repair in Ghana, further expansion of the surgical work force is required. There are two practicing MDs for every practicing specialist doctor in Ghana [38]. If task sharing with MDs is deemed clinically safe for patients in Ghana, it would be both practical and cost-effective to include both surgeons and MDs in this human resource expansion. Since the current distribution of doctors and surgeons is concentrated in certain regions and urban areas [38,39], redistribution of staff may be necessary in order to provide surgical services for the entire country’s population.

The low doctor to population ratio [38] as well as the high workload among the existing health workers [39], is a barrier to immediate or rapid training of already available potential surgical providers. This is fuelled by inadequate training, equipment and supplies [39], despite the abundant number of health facilities in the country [40].

Barriers to patient engagement, such as low levels of health literacy and capacity to pay for the required services, could also hinder the implementation of surgical service expansion. In Ghana, the National Health Insurance Scheme, with an annual premium of about USD 1.17 to7.8, covers a large part of the cost for hernia repair [41]. Still, there is a certain out-of-pocket payment patients need to bear (about USD 37.4 for the insured and USD 98.37 for the uninsured) which can be impediment to access to surgical care, especially for patients from low socio-economic groups.

While there has been a national non-communicable disease (NCD) policy adopted in Ghana since 2012, priority is still largely given to communicable diseases due to high prevalence and interest of international donors [42]. The government allocates a small budget for NCDs [42,43], including surgical conditions, and global aids and grants are earmarked for specific programs. Thus, political commitment and secure funding support are crucial in scaling up surgical care for inguinal hernia [44].

Based on the latest national health account published (2005–2010) in Ghana, total annual health expenditure is projected to be USD 1,938 million in 2020 [45], of which the estimated government spending is USD 695 million according to the reported percent of 38.89% from the World Bank [46]. This study estimates that expanding surgical service delivery with surgeons and MDs would cost USD 110 million if about half (56%) of the total backlog of 1.4 million inguinal hernias are to be repaired by 2030, and USD 218 million to eliminate the entire backlog by 2030. The annual cost in the first scenario is equivalent to 1.4% of the estimated annual government health expenditure and that in the second scenario is of 2.8%. This will include cost for training and potential renovations of facilities. The training cost used in the current analysis was assumed for surgical providers who were already performing inguinal hernia repairs. If novices are to be trained, the costs will increase.

Given the average provider productivity of 50 or 200 hernia repairs per year, an additional 1,200 to 1,250 providers would need to be trained. This is a significant proportion of the total number of MDs in Ghana which was reported to be 2,181 in 2019 with an annual increase of about 100 [38]. Additional reduction in the total cost can be brought about by increasing the number of providers per facility and their annual productivity in hernia repair.

It is possible that an initiative targeting inguinal hernia will lead to positive spill-over effects, such as increasing surgical capacity and strengthening the health care system of the country. Societal costs and economic productivity loss associated with untreated inguinal hernia would be mitigated. Economies of scale can also be expected from the large-scale availability of the service and marginal returns from the capital investment in training, infrastructure and equipment.

In contrast, the service expansion has potential opportunity costs of forgoing health benefits from sharing the resources which otherwise could be used for other healthcare services. Yet, as revealed by previous studies [47,48] and the results from the current study, cost and cost-effectiveness of inguinal hernia repair compare favourably with treatment of HIV/AIDS and TB, and childhood immunization. Inguinal hernia repair should be prioritized alongside other public health interventions.

Expanding certain health services, such as hernia repair, in Ghana will be met with many challenges. Among them are insufficient human resources, inadequate budgetary allocation to health and poor leadership and management. Furthermore, the expansion of one specific service will have implications for others. Priority setting will require careful planning and balancing of gains and costs from different interventions against one another. Subsequent implementation will meet many barriers, some possible to overcome, others less so. This article has illustrated a rationale for including hernia repair in forth-coming discussions on how to best use available resources allocated to health care in Ghana. In reality, achieving good health for all will require scaling up of several health care interventions as well as prevention strategies. Dedicating a very large proportion of the doctors clinically active in Ghana to hernia surgery is not feasible. More doctors will need to be trained and retained in the country in order to meet all health-related needs, including hernia surgery, in the population.

The study has several strengths. First, the main source of data was taken from a controlled prospective cohort study along with the assumptions based on published scientific evidence. Secondly, the study took account of salient clinical events, such as risk of recurrence and risk of contralateral hernia, known to have significant influence on the long-term course of disease.

The main limitation of the current study is that it does not take into consideration the full cost of training for MDs and surgeons. In order to provide scaled up surgical services for groin hernia as well as other important surgical conditions, more MDs and surgeons need to be educated and trained. Allocating resources to a specific condition such as groin hernia is complex as no MD or surgeon will carry out hernia repairs only.

The results of the study refer to costs and cost-effectiveness of expanding surgical capacity for groin hernia repair in men. Future studies should investigate the cost-effectiveness of repairing hernia in children and women. Hernias in these two groups are less common than in men but still constitute significant population-level health problems [8].

## Conclusions

Scaling up of hernia repair is highly cost-effective, and by training 1,200 to 1,250 surgical providers to perform mesh inguinal hernia repair, the backlog of groin hernia in Ghana can be eliminated by 2030. The findings highlight a substantial and affordable reduction of the disease burden through accelerating elective inguinal hernia repair to eliminate the backlog of hernia surgery in the currently under-served Ghanaian population. Further research is warranted to explore how to overcome health system barriers preventing expansion of essential and cost-effective health services.

## Supporting information

S1 Fig Service expansion of inguinal hernia repair with annual cap of USD 10 million in Ghana. The line graph displays estimated annual cost (in USD millions) for service expansion of inguinal hernia repair, with annual cap of USD 10 million, and associated number of people with untreated hernia (in thousands) in Ghana from year 2020 to 2030.
(TIF)

S2 Fig Health gains from the service expansion with annual cap of USD 10 million in Ghana. *DALY* disability-adjusted life year. The line graph displays number of DALYs averted (in thousands) expected from service expansion of inguinal hernia repair with annual cap of USD 10 million in Ghana from year 2020 to 2030.
(TIF)

S3 Fig Eliminating the backlog of inguinal hernia among adult men in Ghana by 2030. The line graph displays estimated annual cost (in USD millions) for eliminating the backlog of inguinal hernia among adult men and associated number of people with untreated hernia (in thousands) in Ghana from year 2020 to 2030.
(TIF)

S4 Fig Health gains from eliminating the backlog of inguinal hernia among adult men in Ghana. *DALY* disability-adjusted life year. The line graph displays number of DALYs averted (in thousands) expected from eliminating the backlog of inguinal hernia among adult men in Ghana from year 2020 to 2030.
(TIF)

S5 Fig Deterministic sensitivity analysis on the backlog of Ghanian men with untreated hernias by 2030. The tornado chart displays percent decrease or increase in the backlog of Ghanian men with untreated hernias by year 2030 from variations of inputs to the budget impact analysis.
(TIF)

S6 Fig Deterministic sensitivity analysis on the total cost of eliminating the backlog of inguinal hernias among Ghanaian men by 2030. The tornado chart displays percent decrease or increase in the total cost of eliminating the backlog of inguinal hernias among Ghanian men by year 2030 from variations of inputs to the budget impact analysis.
(TIF)
