# Supplementary figures and images for: Economic evaluation of expanding inguinal hernia repair among adult males in Ghana
Source: PLOS Glob Public Health. 2022 Apr 4;2(4):e0000270. doi: 10.1371/journal.pgph.0000270 (PMC10022161; doi:10.1371/journal.pgph.0000270)

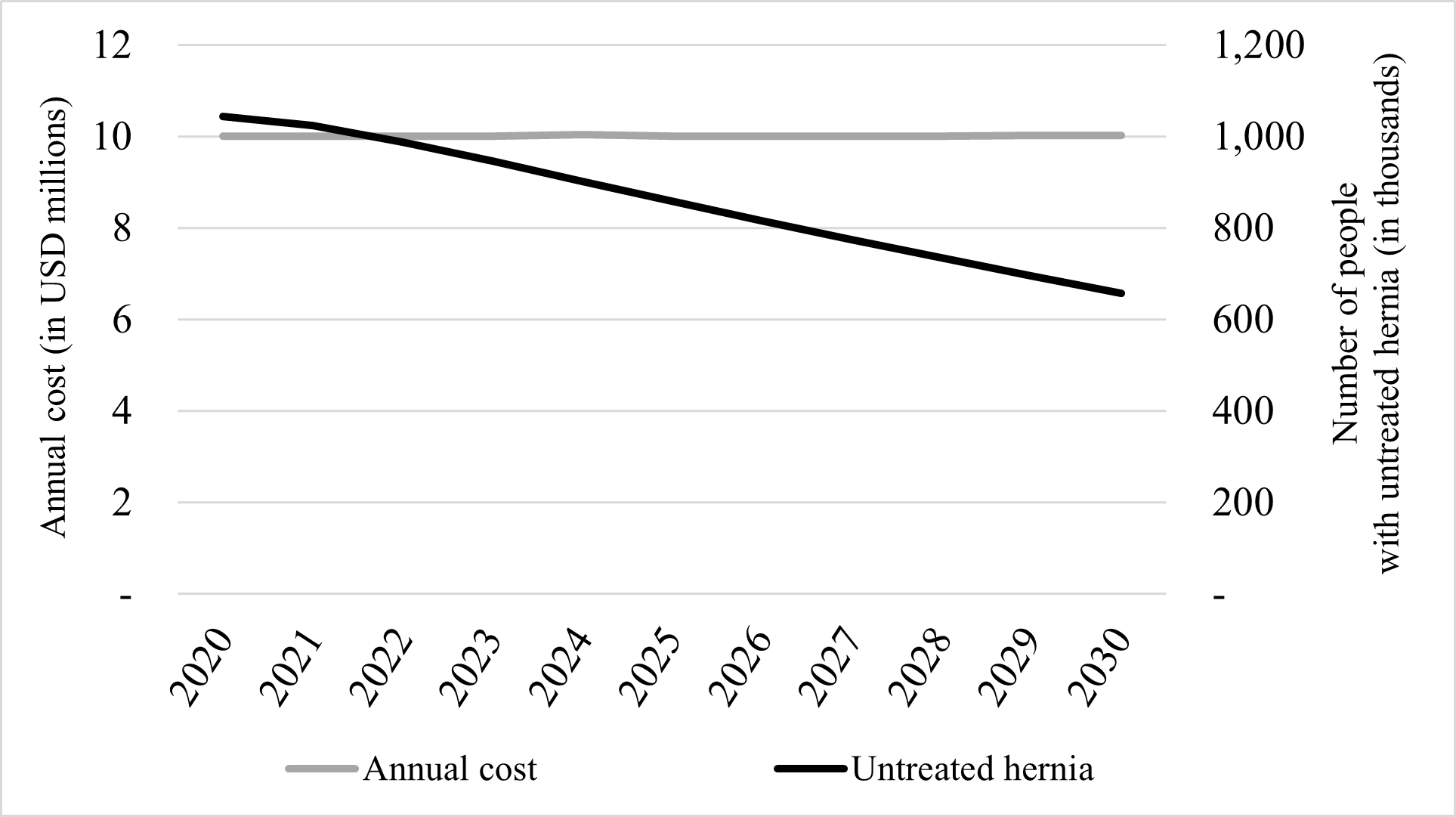

Supplement: S1 Fig — The line graph displays estimated annual cost (in USD millions) for service expansion of inguinal hernia repair, with annual cap of USD 10 million, and associated number of people with untreated hernia (in thousands) in Ghana from year 2020 to 2030. (TIF) [file pgph.0000270.s001.tif]

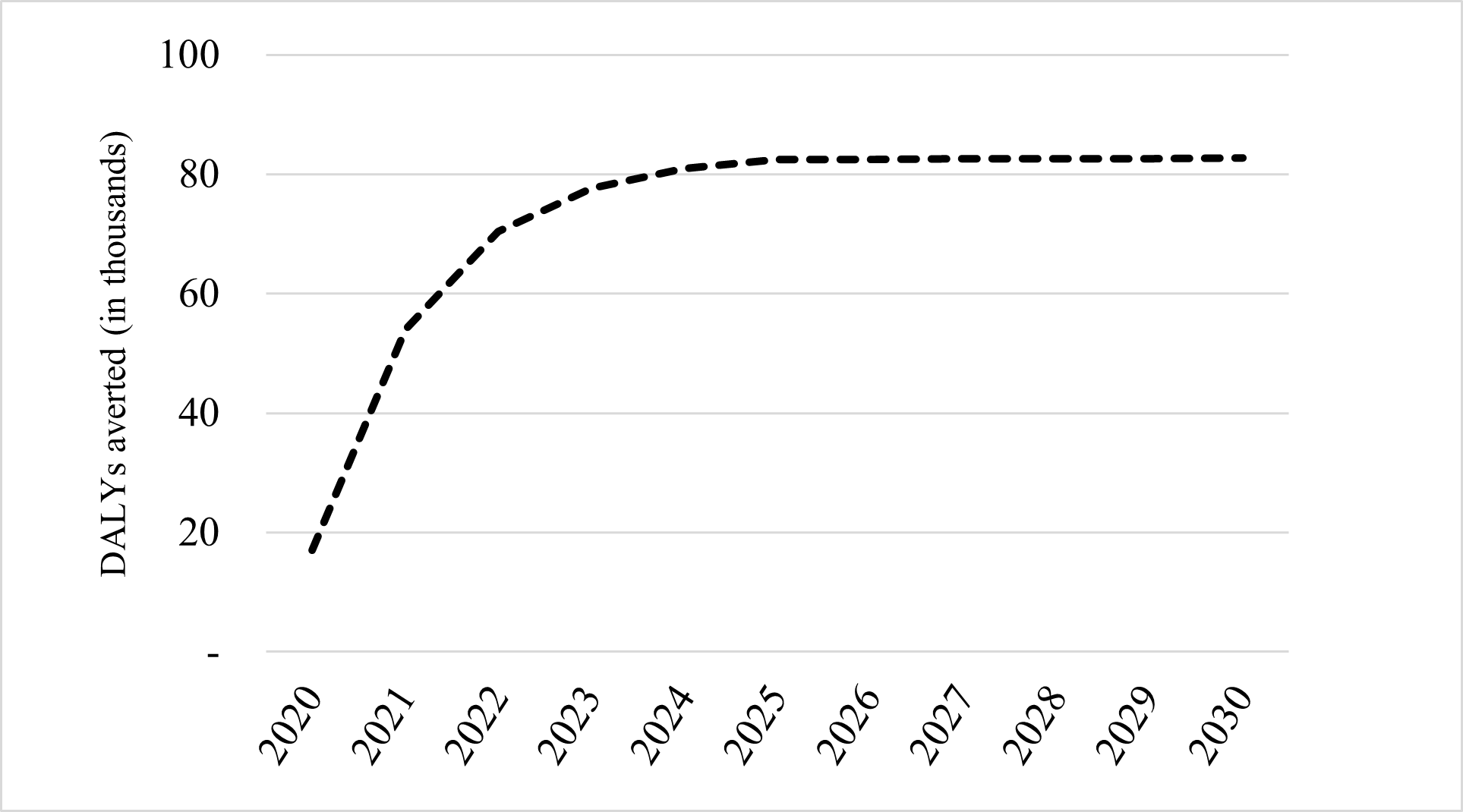

Supplement: S2 Fig — DALY disability-adjusted life year. The line graph displays number of DALYs averted (in thousands) expected from service expansion of inguinal hernia repair with annual cap of USD 10 million in Ghana from year 2020 to 2030. (TIF) [file pgph.0000270.s002.tif]

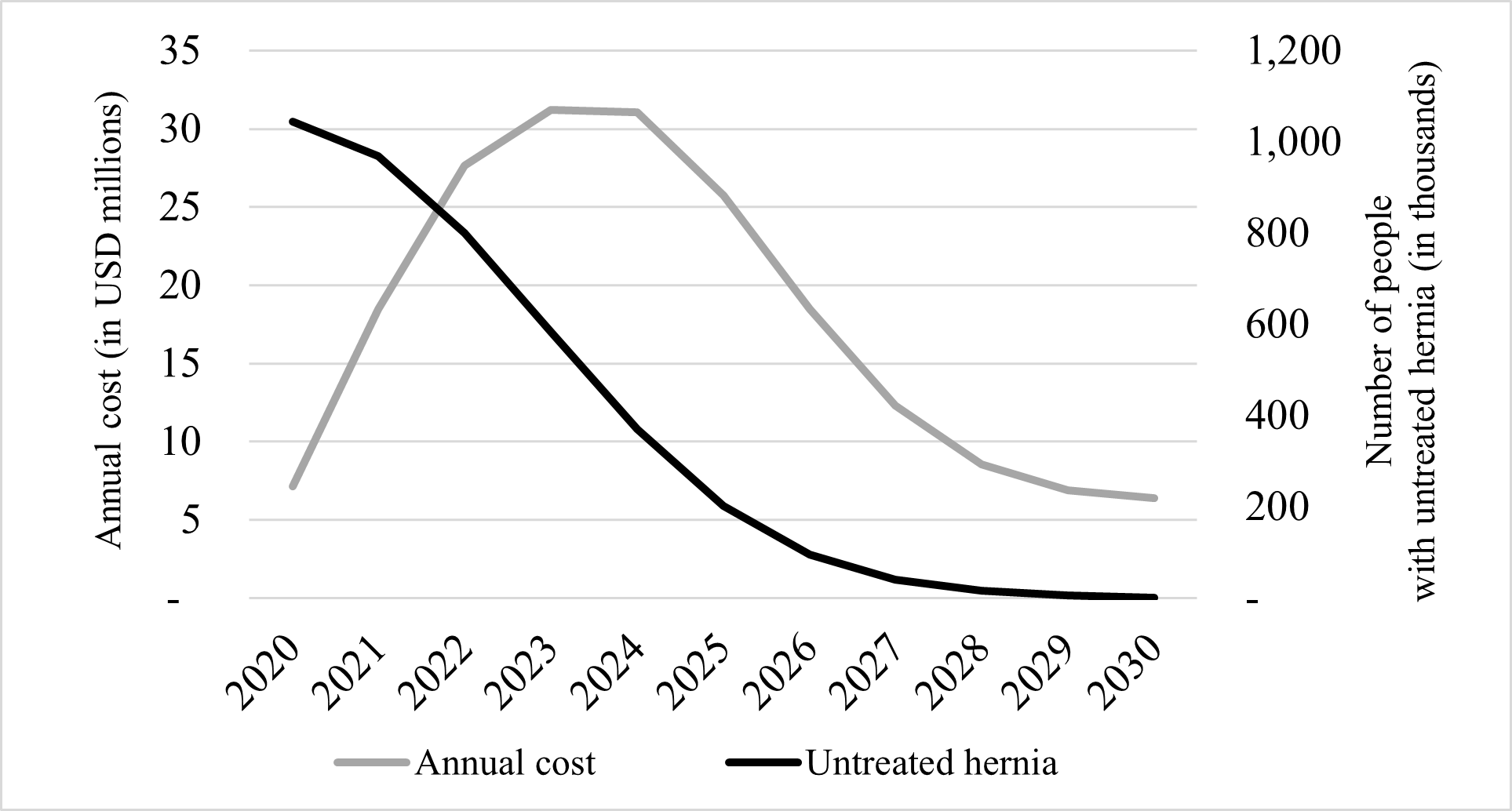

Supplement: S3 Fig — The line graph displays estimated annual cost (in USD millions) for eliminating the backlog of inguinal hernia among adult men and associated number of people with untreated hernia (in thousands) in Ghana from year 2020 to 2030. (TIF) [file pgph.0000270.s003.tif]

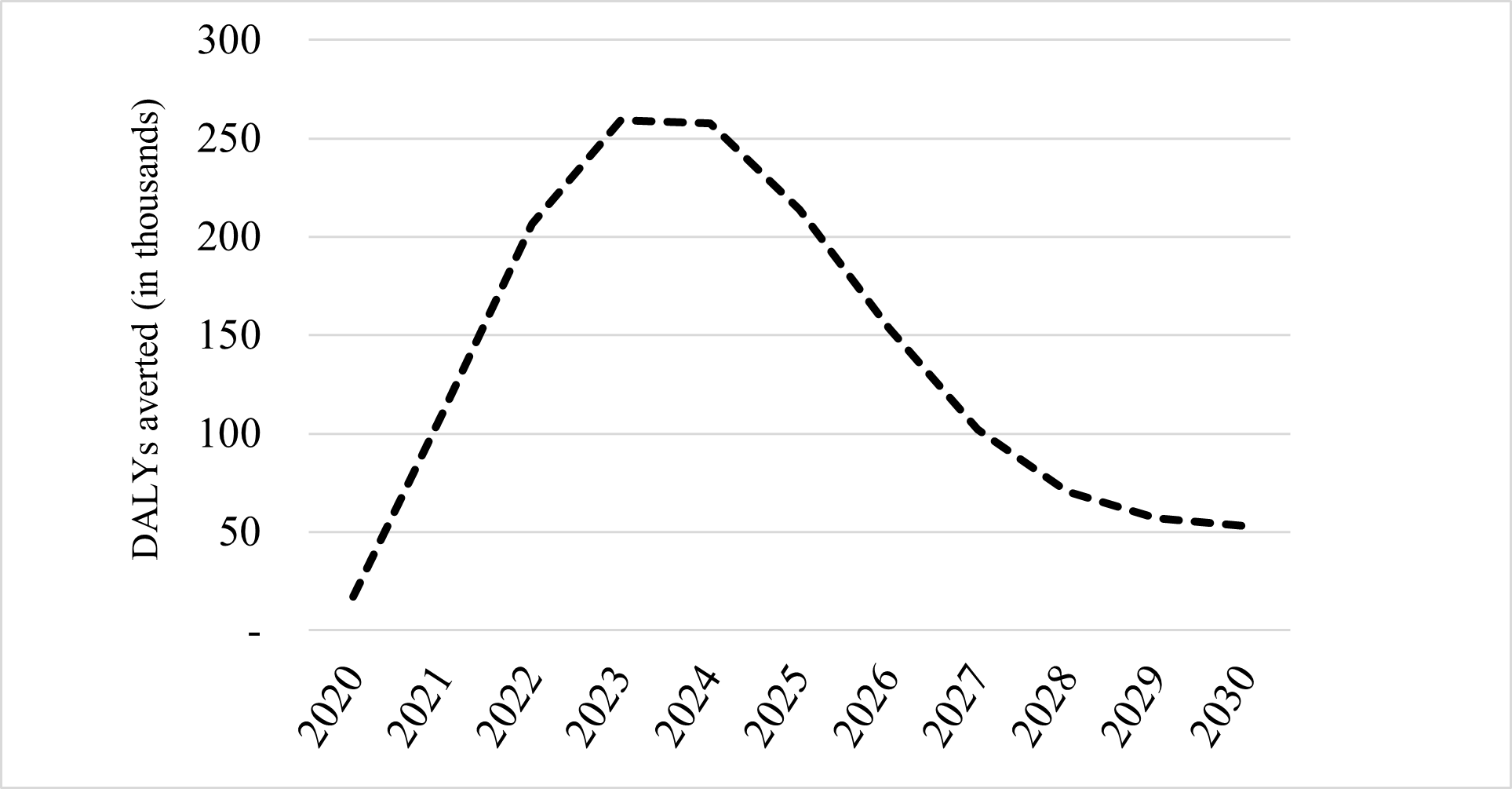

Supplement: S4 Fig — DALY disability-adjusted life year. The line graph displays number of DALYs averted (in thousands) expected from eliminating the backlog of inguinal hernia among adult men in Ghana from year 2020 to 2030. (TIF) [file pgph.0000270.s004.tif]

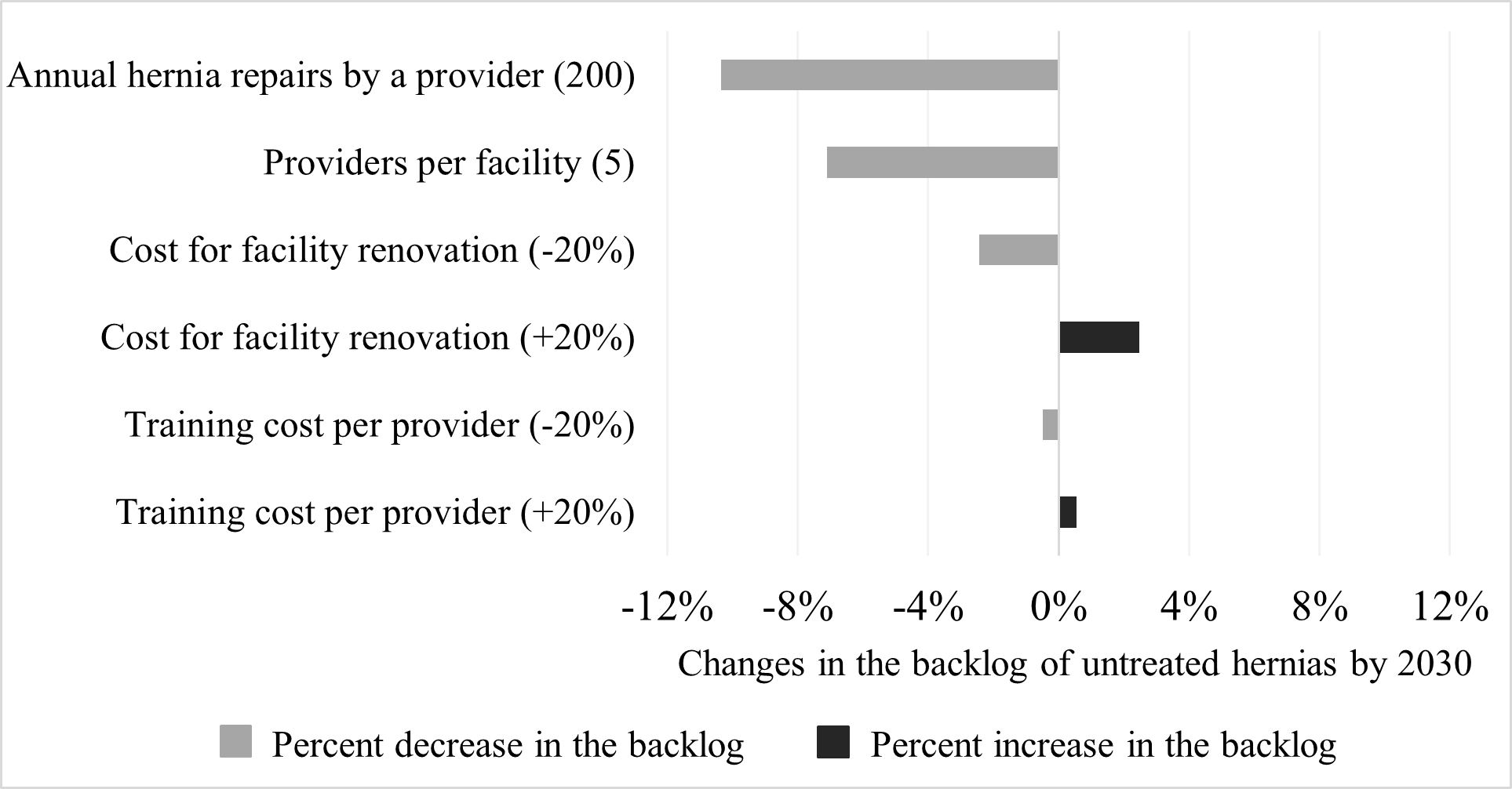

Supplement: S5 Fig — The tornado chart displays percent decrease or increase in the backlog of Ghanian men with untreated hernias by year 2030 from variations of inputs to the budget impact analysis. (TIF) [file pgph.0000270.s005.tif]

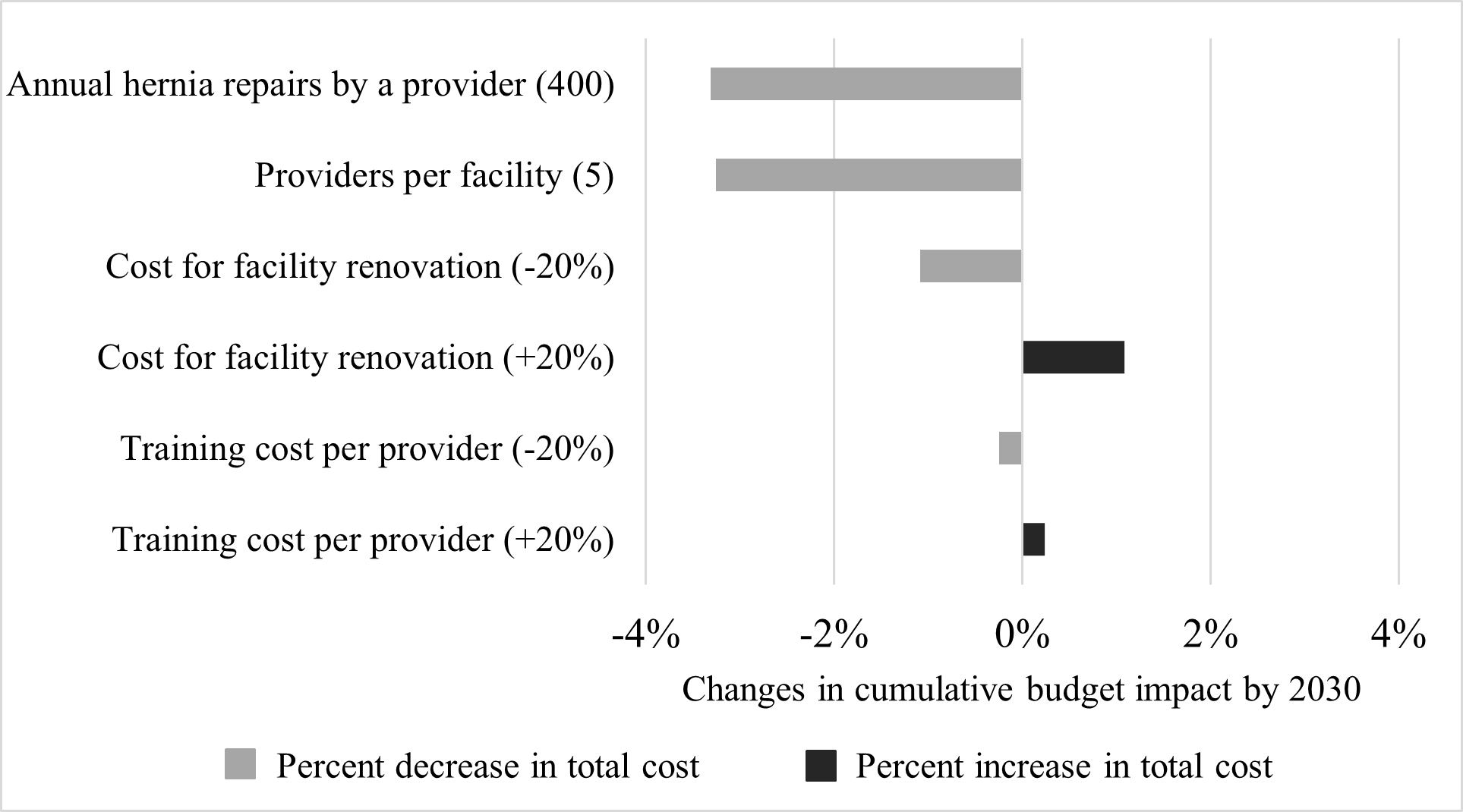

Supplement: S6 Fig — The tornado chart displays percent decrease or increase in the total cost of eliminating the backlog of inguinal hernias among Ghanian men by year 2030 from variations of inputs to the budget impact analysis. (TIF) [file pgph.0000270.s006.tif]
